# A possible role for autoimmunity through molecular mimicry in alphavirus mediated arthritis

**DOI:** 10.1038/s41598-019-55730-6

**Published:** 2020-01-22

**Authors:** Siva Sai Krishna Venigalla, Sowmya Premakumar, Vani Janakiraman

**Affiliations:** Department of Biotechnology, Bhupat and Jyoti Mehta School of Biosciences, Indian Institute of Technology Madras, Chennai, 600036 India

**Keywords:** Computational biology and bioinformatics, Autoimmunity

## Abstract

Alphaviral infections are foremost in causing debilitating clinical outcomes in humans characterized by rheumatic arthritis like conditions. Though the presence of virus in joints and associated inflammation has been implicated as one of the reasons for the acute and chronic polyarthritis post alphaviral infections, the basis for rheumatic like outcomes is not clear. Through an *in silico* analysis, we have investigated the possibility of an autoimmune process mediated through molecular mimicry in alphaviral infection induced pathogenicity. Interestingly, sequence alignment of the structural polyproteins belonging to arthritogenic alphaviruses revealed conserved regions which share homology with human proteins implicated in rheumatoid arthritis (RA). These conserved regions were predicted to exhibit binding to HLA class II alleles, showcasing their potential to incite T cell help. Molecular docking of the viral peptide and the corresponding homologous region in the human protein onto HLA-DRB1 revealed strong similarities in their binding patterns. Linear and conformational B cell epitope prediction analyses showed that these potential mimics have high propensity to elicit an efficient B cell response. We thus propose that the origin of polyarthritis post-arthritogenic alphaviral infections may also be mediated through a hitherto unknown autoimmune response due to the presence of cross-reactive epitopes between viral and human proteins.

## Introduction

Alphaviruses belonging to the group IV togoviridae family are positive sense, single stranded RNA viruses. These enveloped viruses are classified as old world and new world. While new world viruses are encephalitogenic, members of the old world are known to induce polyarthritis^1^. The members of the old world viruses include Chikungunya Virus (CHIKV), Ross River Virus (RRV), Mayaro Virus (MAYV), O’nyong nyong virus (ONV), Semiliki Forest Virus (SFV) and the Barmah Forest Virus (BFV). These mosquito transmitted viruses are globally distributed and are known to cause acute febrile illnesses, malaise, maculopapular rashes, myalgia, and severe arthralgia in humans^2–5^. Most often the infection remains endemic, but some viral strains are also associated with large epidemics such as the Chikungunya virus (CHIKV) out-break which was spread across 40 countries with 1.4–6.5 million reported cases globally^6^. Several studies also report a simultaneous increase in alpha virus associated arthritis lasting even after decrease in viral load^7^.

While there is a resurgence of alphavirus associated arthritis cases, the knowledge about the molecular level events during infections involved in these conditions and about the direct cause and effect of the phenomenon is very sparse. For instance, macrophages, natural killer cells, CD4+ and CD8+ T lymphocytes have been shown as the main components of the inflammatory cellular infiltrate in animal models of CHIKV and RRV infections^8–10^. Production of a broad range of pro-inflammatory cytokines (IL-6, TNF, IFN-α/β, and IFNγ) and chemokines (MCP-1 and RANTES) post alpha viral infections has also been reported by *Lidbury et al*.^11^. Interestingly, a comparative study between CHIKV infection induced gene expression and rheumatoid arthritis induced gene expression changes in mouse models showed similarity in induced cytokine (TNF, IL-8, IL-15, IFN-γ, GM-CSF and lymphotoxin B) profiles^12^. Another study by Morrison *et al*.^13^, demonstrated the presence of activated complement products in the serum of RRV infected mice, drawing similarities with the pathogenesis of rheumatoid arthritis^13^.

Resemblance between proteins of pathogens and host components has been examined as a trigger for many autoimmune diseases^14^. In these scenarios, initiation of autoimmune disease by an infectious pathogen is hypothesized to involve immune recognition of self-peptides by way of “molecular mimicry”^15^ thereby activating autoreactive T cells and generation of autoantibodies. Previous studies^16,17^ hypothesized that microbial antigens like pulD from Klebsiella sp., nuclear antigen-1 from Epstein-Barr virus and OSP-A from Borrelia sp. may have a possible association with autoimmune diseases like ankylosing spondylitis, Systemic Lupus Erythematosus (SLE) and Lyme arthritis, respectively. A significant sequence similarity between P2-C protein of Coxsackie virus and glutamate decarboxylase of humans has been proposed as a trigger for Type 1 diabetes^18^. The similitude in clinical manifestation of an alphaviral infection induced arthralgia and rheumatoid arthritis at both phenotypic and molecular levels suggests that alphaviral infections could be a causative link for rheumatoid arthritis.

Overarching goal of this study is to explore the possibility of existence of peptides in alphaviruses which may set off an autoimmune response resulting in rheumatoid arthritis like symptoms in alphavirus infected patients. Structural polyproteins of the alphaviruses were scanned for the presence of conserved stretches of amino acids that might share homology with sequences in human proteins implicated in rheumatoid arthritis. These conserved regions were validated *in silico* both at sequence and structural level for their immunogenic potential in terms of their ability to act as T and B cell epitopes. Overall, our results posit that polyarthritis associated with alpha viral infections may also involve an autoimmune component due to cross reactivity between viral epitopes and host proteins.

## Results

**Figure 1:**
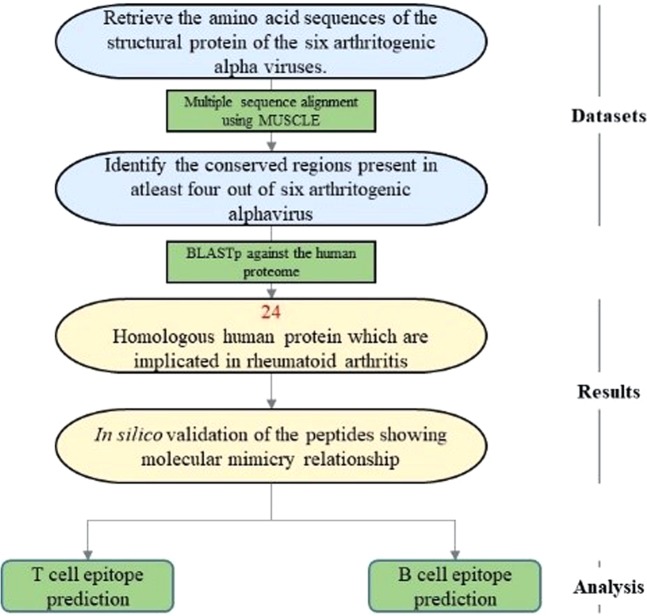
Computational pipeline for the prediction of possible induction of rheumatoid arthritis through molecular mimicry post alphaviral infection.

Arthritogenic alpha viruses such as Chikungunya virus (CHIKV), Ross river virus (RRV), Semiliki forest virus (SFV), Mayaro virus, O’nyong-nyong virus (ONV) and Barmah forest virus (BFV) are generally associated with rheumatic diseases in humans, primarily characterized by polyarthralgia and polyarthritis. Although most patients recover within few weeks, the clinical conditions last for 6 months to more than 3 years^6^. However, molecular mechanisms contributing to this pathology are not very well understood. In the present study, we show a possible role of autoimmune process mediated through molecular mimicry as a likely cause for arthritic like conditions post alphaviral infections. The overall workflow of the study is depicted in Fig. 1.

### Multiple sequence alignment reveals conserved regions in the structural polyproteins of arthritogenic alpha viruses

**Figure 2:**
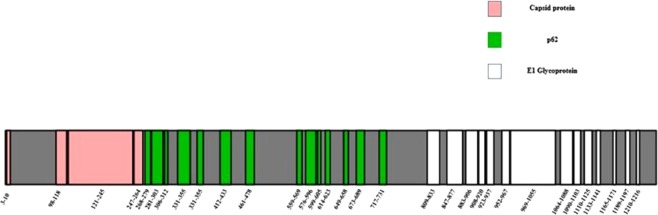
Domain diagram of the regions conserved in the structural polyprotein of the alphaviruses. The regions have been depicted using Chikungunya virus (strain S27 African prototype) structural polyprotein as a representative model.

Alphaviral infections share a common feature of inducing RA like conditions, but the commonality in the alphaviruses leading to such a rheumatic outcome is not known. We hypothesized that the commonality could lie in the structural polyprotein of these viruses. Therefore, we have analyzed the structural polyproteins of six arthritogenic alpha viruses for the presence of conserved regions amongst them. As described in the methods section, amino acid sequences of the structural polyproteins of CHIKV, RRV, SFV, ONV, BFV and Mayaro virus were retrieved from UniProt database and subsequently multiple sequence alignment was performed using MUSCLE. Conserved regions identified are shown in Supplementary Fig. 1. The positions of the conserved regions have been depicted in Fig. 2 using the structural polyprotein of the Chikungunya virus as a representative model.

The structural polyproteins of arthritogenic alphaviruses are about 71% identical at the primary (amino acid sequence) level. A total of 41 conserved regions were identified in the structural polyproteins, with E1 glycoprotein region exhibiting highest conservancy with 18 conserved regions, followed by p62 and capsid proteins harboring 15 and 8 conserved regions respectively.

### Conserved regions across alpha viruses share homology with human proteins implicated in arthritis

To deduce the possibility of autoimmune reactions involved in mediating post alphaviral polyarthralgia through the presence of molecular mimics, we performed a sequence similarity search between the conserved regions among arthritogenic alpha viruses and the human proteome using the standard BLASTp program. Each of the 41 conserved regions was scanned against the human proteome. Very interestingly, 24 regions showed varying degrees of homology (52.7% to 100%) with human proteins that have been implicated in arthritis like conditions in humans (Table 1).

**Table 1:** List of human proteins sharing homology with conserved regions of structural polyproteins of alphaviruses and shown to contribute to rheumatoid arthritis-like-condition.

Viral peptide	Human peptide	Human protein	UniProt ID	% similarity
FIPTQTFY	FIPT	Ryanodine receptor 1	P21817	100%
KGRVVAIVLGGANEGARTALSVVTW	ARTALS	Interleukin-17 receptor C	Q8NAC3	100%
TSAPCTITGTMGHFILARCPKG	ITGTM	Monocyte differentiation antigen CD14	P08571	100%
**RKGKIHIPFPLANVTCMVPKA**	**PLANVT**	**Alpha-1B-glycoprotein**^44^	P04217	100%
PTVTYGK	PTVTYG	Intercellular adhesion molecule 1	P05362	100%
**PTVTYGK**	**TVTY**	**Low-density lipoprotein receptor-related protein 2**^45^	P98164	100%
CGTAEC	TAEC	Interleukin-23 receptor	Q5VWK5	100%
PDYSCKVFTGVYPFMWGGAYCFCD	YCFC	Platelet glycoprotein 4	P16671	100%
PDYSCKVFTGVYPFMWGGAYCFCD	SCKVF	Glucocorticoid receptor	P04150	100%
PDYSCKVFTGVYPFMWGGAYCFCD	SCKVF	Androgen receptor	P10275	100%
FSTALAS	STALA	Leukocyte cell-derived chemotaxin-2	O14960	100%
PPCIPCCYEKEPEETLRMLEDNV	YEKEPGEE	Interleukin-1 receptor accessory protein	Q9NPH3	87.5%
**YSGGRFTIPTGAGKPGDSGRPIFDN**	**GKPGDDG**	**Collagen alpha-1(II) chain**^46,47^	P02458	86%
RKGKIHIPFPLANVTCMVPKA	PLANVIC	TNF receptor-associated factor 6	Q9Y4K3	85.7%
**YNMDYPPFGAGRPGQFGDIQSRTPE**	**IASRTPE**	**Annexin A5**^48^	P08758	85.7%
DIPDAAFTRVVDAP	DAAFTRI	Vitronectin	P04004	85.7%
PGYYQLL	GYYDLL	Ryanodine receptor 1	P21817	83.3%
DGTLKIQVSLQIG	NGTLKI	T-cell surface antigen CD2	P06729	83.3%
TSAPCTITGTMGHFILARCPKG	SAPCTV	Integrin alpha-IIb	P08514	83.3%
KWQYNSPLVPR	DSPLVP	Leptin receptor	P48357	83.3%
KWQYNSPLVPR	SPIVPR	Peptidoglycan recognition protein 1	O75594	83.3%
**QAPSGFKYWLKE**	**SGFIYW**	**Low-density lipoprotein receptor-related protein 2**^45^	P98164	83.3%
DGTLKIQVSLQIG	DGTLFKVQV	Beta-sarcoglycan	Q16585	77.8%
MKSDASKFTHEKPEGYYNWHHGAVQ	KSEADKFT	Immunoglobulin superfamily member 6	O95976	75%
YSGGRFTIPTGAGKPGDSGRPIFDN	AGRPGNSG	Macrophage scavenger receptor types I and II	P21757	75%
KFIVGPMSSAWTPFDNKIVVYKGDV	FDNTTVVY	Interleukin-23 receptor	Q5VWK5	75%
SKDVYANTQLVLQRPAAGTVHVPYS	TKLLLQRP	High affinity immunoglobulin gamma Fc receptor I	P12314	75%
APFGCQIATNPVRAMNCAVGNMPIS	ALGQMPIS	Coagulation factor V	P12259	75%
RKGKIHIPFPLANVTCMVPKA	PMVNVTC	MHC class I polypeptide-related sequence B	Q29980	71.4%
KFIVGPMSSAWTPFDNKIVVYKGDV	WIPFQNK	Lymphocyte antigen 75	O60449	71.4%
KPGRRERMCMKIENDCIFEVK	ENECFFE	Protocadherin-9	Q9HC56	71%
**SKDVYANTQLVLQRPAAGTVHVPYS**	**QSPAAGTVQGRVP**	**Cartilage intermediate layer protein 1**^49,50^	O75339	69.2%
FIPTQTFY	PTEKFY	Complement C3	P01024	67%
CGTAEC	CGVAQC	Integrin alpha-V	P06756	66.6%
RKGKIHIPFPLANVTCMVPKA	IPIPLA-VITTCIV	Lymphocyte function-associated antigen 3	P19256	64. 3%
NADLAKLAFKRSSKYDLECAQIPVH	YDLDCPTAPIP	TNF receptor-associated factor 6	Q9Y4K3	64%
FIPTQTFY	FISTQQQVTF	High affinity immunoglobulin epsilon receptor subunit alpha	P12319	60%
PGYYQLL	GYRETRYQLL	N-alpha-acetyltransferase 16	Q6N069	60%
PTVTYGK	PTVTTGSGYG	V-set and immunoglobulin domain-containing protein 4	Q9Y279	60%
RKGKIHIPFPLANVTCMVPKA	IPY-LATDVTCVGP	Toll-like receptor 7	Q9NYK1	57.1%
**EGLEVTWGNN**	**DGLDVSW**	**Chitinase-3-like protein 2**^51^	Q15782	57.1%
**FIPTQTFY**	**IPSRSFY**	**Cartilage intermediate layer protein 1**^49,50^	O75339	57%
**MKSDASKFTHEKPEGYYNWHHGAVQ**	**ANKFTETNPQVYY**	**Low-density lipoprotein receptor-related protein 2**^45^	P98164	54%
AYEHVTVIPNTVGVPYKTLVNRPGY	PATVGVTQPY–LDRLGY	Cartilage intermediate layer protein 2	Q8IUL8	52.6%

Human proteins highlighted in bold are experimentally proven to have antibodies developed against them in patients diagnosed with rheumatoid arthritis.

### Potential mimic regions harbor HLA binding motifs

Human leukocyte antigen (HLA) is the most widely distributed molecule with high level of polymorphism and are the major governing factors in initiating cellular immune responses. It is thus important that immunogenic peptides bind to multiple HLA alleles to elicit a strong immune response. The 24 conserved regions of the structural polyprotein (refer Table 1) were analyzed for HLA class II binding ability using propred II server. The nonameric peptides generated from each conserved region were scanned against 51 HLA alleles available in the ProPred II server. The number of nonameric peptides generated from each of 24 conserved regions and the number of peptides predicted to bind to different alleles is summarized in Table 2. Peptides generated from 19 of the conserved regions bound to at least one HLA allele, while the peptides from the remaining 5 of the conserved regions did not bind to any of the alleles.

**Table 2:** HLA binding profiles of potential mimics from the alpha viral structural proteins. A total of 51 HLA class II alleles were used for the study.

Peptide	Peptide Length (aa)	Binding profile -HLA Class II		
		Nonamers generated	Nonamers bound	% binding
PPCIPCCYEKEPEETLRMLEDNV	23	15	0	0
PTVTYGK	7	5	0	0
EGLEVTWGNN	10	2	0	0
QAPSGFKYWLKE	12	4	0	0
DIPDFTRVVDAP	14	6	0	0
KPGRRERMCMKIENDCIFEVK	21	13	3	6
NADLAKLAFKRSSKYDLECAQIPVH	25	17	1	6
MKSDASKFTHEKPEGYYNWHHGAVQ	25	17	1	6
YSGGRFTIPTGAGKPGDSGRPIFDN	25	17	1	6
YNMDYPPFGAGRPGQFGDIQSRTPE	25	17	2	12
SKDVYANTQLVLQRPGTVHVPYS	25	17	2	12
APFGCQIATNPVRAMNCAVGNMPIS	25	17	2	12
RKGKIHIPFPLANVTCMVPKA	21	13	2	15
KGRVVAIVLGGANEGARTALSVVTW	25	17	3	17
KFIVGPMSSAWTPFDNKIVVYKGDV	25	17	3	18
PDYSCKVFTGVYPFMWGGAYCFCD	24	16	3	19
PGYYQLL	7	5	1	20
DGTLKIQVSLQIG	13	5	1	20
FSTALAS	7	5	1	20
TSAPCTITGTMGHFILARCPKG	22	14	3	21
AYEHVTVIPNTVGVPYKTLVNRPGY	25	17	4	24
CGTAEC	12	4	1	25
KWQYNSPLVPR	11	3	1	33
FIPTQTFY	8	5	2	40

**Figure 3:**
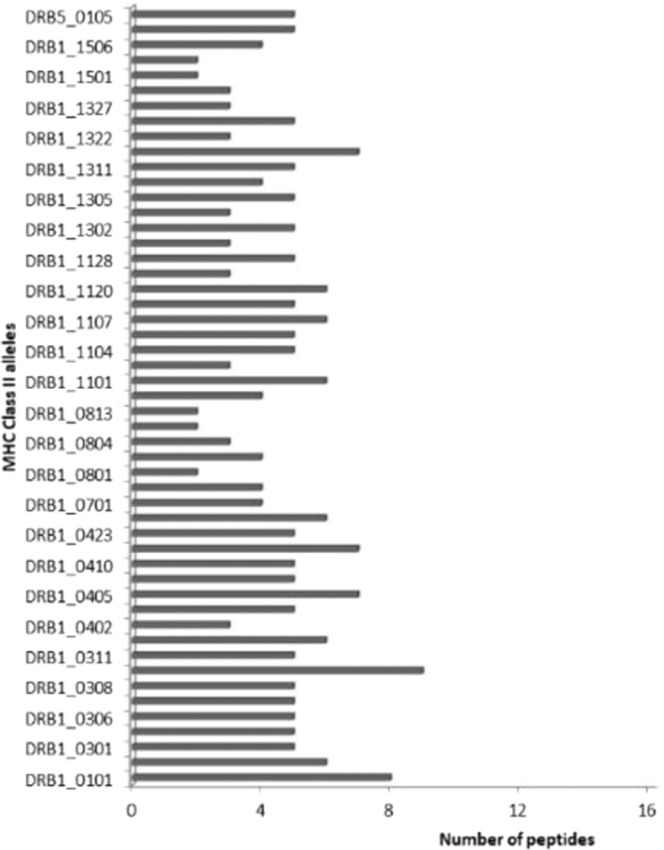
Number of conserved regions from alpha virus structural proteins binding to individual class II alleles as predicted by ProPred II server. Alleles that exhibited binding to more than 3 peptides are represented out of the 51 alleles studied.

As illustrated in Fig. 3, maximum numbers of nonamers were found to be recognized by the allele HLA DRB1_0308, which bound to nine of the conserved region peptides. DRB1_0101 bound to eight and DRB1_1321 bound to seven, DRB1_0102, 0401, 0426, 1101, 1107 and 1120 bound to six conserved region peptides respectively.

**Figure 4:**
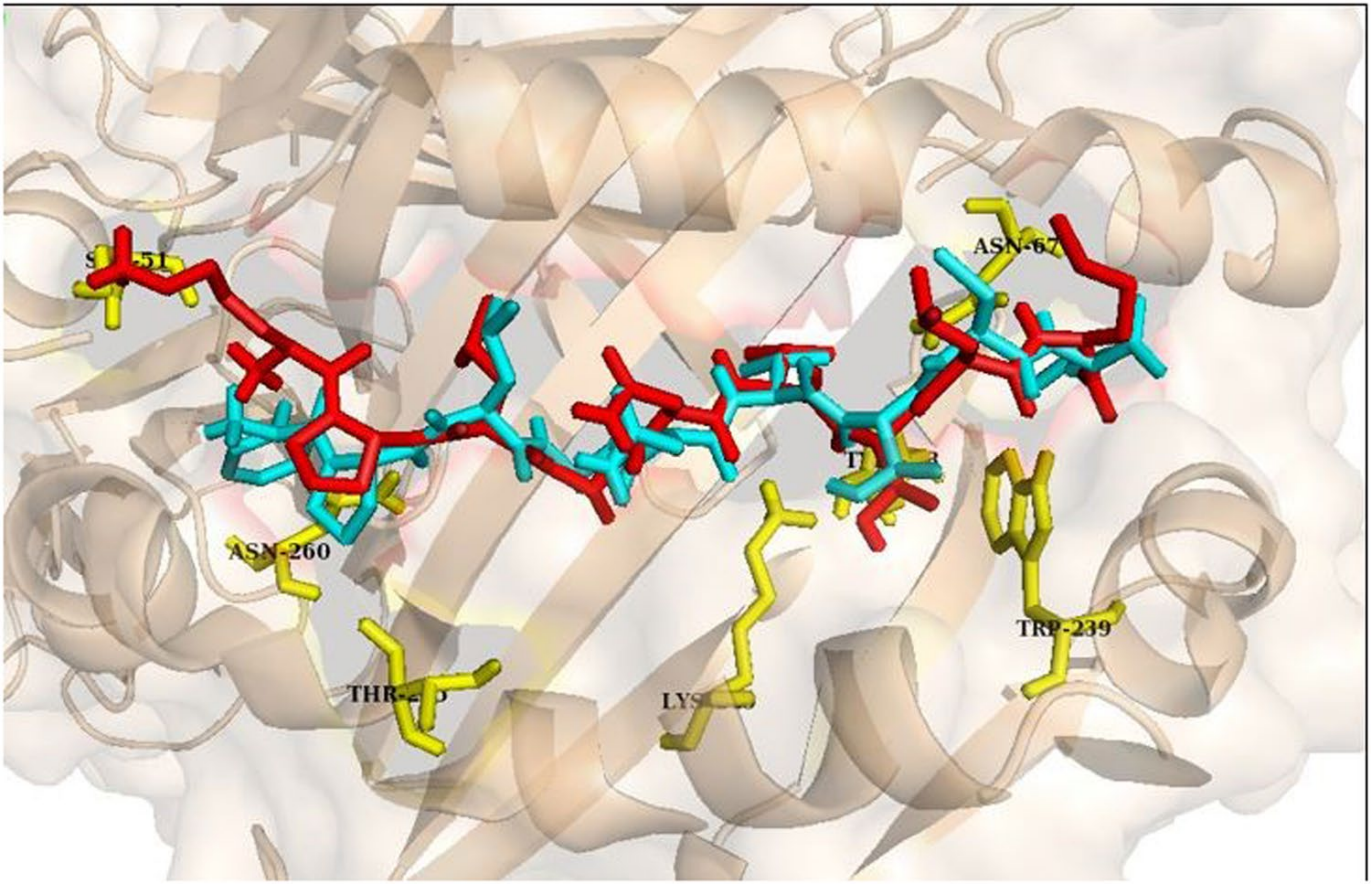
A Structural representation of the peptide-MHC complex generated by docking of the peptide FPLANVTCM from the alpha virus structural polyproteins (cyan) and the peptide KPLANVTLM from the alpha 1-B glycoprotein of human (red) into the binding groove of HLA-DRB1. Both the peptides exhibit similar fit and interactions with the amino acids (yellow) in the binding pocket of HLA.

Since the quality of immune response elicited by the mimic peptide in humans should be similar to the one elicited by the viral peptide for an efficient autoimmune response to occur, the structure of the HLA DRII-peptide complex was analyzed. Molecular docking analysis was performed to assess the structural similarity in the binding patterns of the viral peptide and its homologous region in the human protein. The ability of one of the potential conserved regions, FPLANVTCM (viral derived) and its corresponding homologous region KPLANVTLM (human derived) to bind to a class II HLA allele was assessed by docking the peptides in the binding groove of HLA-DRB1, an allele known to predispose individuals to rheumatoid arthritis^19^. The peptides were docked using GalaxyPepDock server and the docked peptides are shown in Fig. 4. The root mean square deviation between the docked peptides shown in Fig. 4 is 0.86 Å.

**Figure 5:**
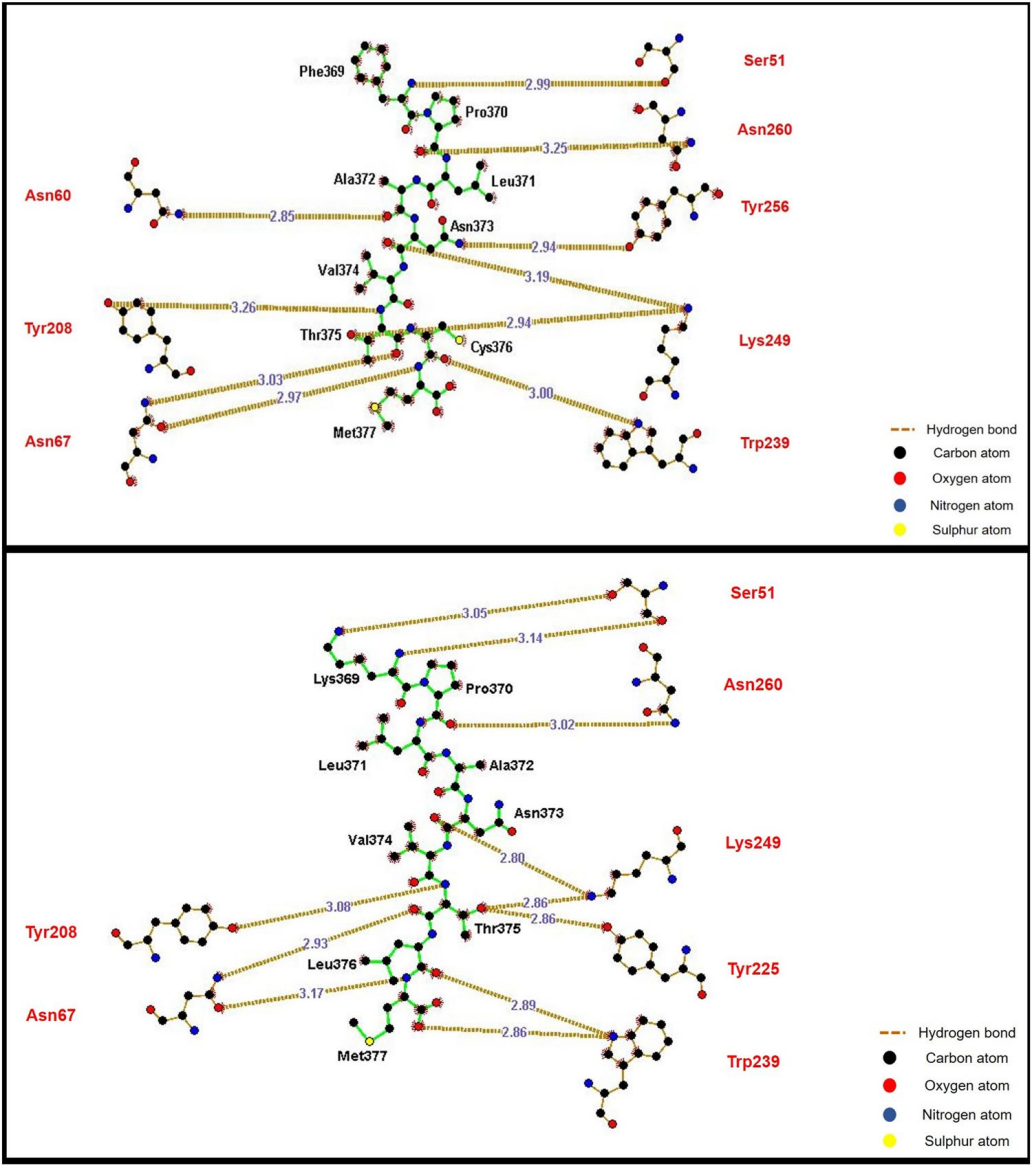
Analysis of molecular interactions of viral peptide docked onto HLA-DRB1 (top) and its homologous region in the human protein docked onto HLA-DRB1 (bottom).

The docked p-MHC complexes were analyzed for the hydrogen bonding interactions between the peptides and HLA-DRB1. The binding interactions between the viral and human peptide with HLA-DRB1 were visualized using LigPlot+. Post docking analysis of the peptides revealed significant interactions with HLA-DRB1. In viral peptide-HLA-DRB1 complex, ten hydrogen bonds within a distance of 3.5 Å suggested the stability of the peptide in the binding groove of the pocket. Similarly, a set of eleven hydrogen bonds were formed between human peptide and HLA-DRB1 within a distance of 3.5 Å. Furthermore, it can be seen in Fig. 5 that the amino acids in the conserved regions of both the peptides form identical hydrogen bonds with the amino acids (Ser51, Asn260, Lys249, Trp235, Asn67, Tyr 208) present in the binding pocket of HLA-DRB1.

### Potential mimics could also act as B cell epitopes

To further strengthen the case of alpha viral induced molecular mimicry, we have analyzed the 24 conserved regions for the presence of both linear and structural B cell epitopes which in turn is a measure of their ability to act as stimulators of B cells and generate an autoimmune reaction against its homologous region in the human protein leading to polyarthralgia. Linear B cell epitope prediction was performed using three different prediction algorithms namely Bepipred linear epitope prediction, Emini surface accessibility scale and Kolaskar and Tongaonkar antigenicity scale. Prediction results are summarized in Table 3.

**Table 3:** Analysis of conserved regions of alpha viruses sharing homology with human peptides (refer Table 1) for their ability to act as B cell epitopes.

Conserved region	Bepipred	ESA	KT
FIPTQTFY	×	×	×
KPGRRERMCMKIENDCIFEVK	✓	✓	✓
NADLAKLAFKRSSKYDLECAQIPVH	×	×	✓
MKSDASKFTHEKPEGYYNWHHGAVQ	✓	×	✓
YSGGRFTIPTGAGKPGDSGRPIFDN	✓	×	×
KGRVVAIVLGGANEGARTALSVVTW	✓	×	✓
PPCIPCCYEKEPEETLRMLEDNV	✓	✓	✓
PGYYQLL	×	×	✓
DGTLKIQVSLQIG	×	×	✓
TSAPCTITGTMGHFILARCPKG	✓	×	✓
KWQYNSPLVPR	✓	✓	✓
RKGKIHIPFPLANVTCMVPKA	✓	×	✓
PTVTYGK	✓	×	×
EGLEVTWGNN	✓	×	×
AYEHVTVIPNTVGVPYKTLVNRPGY	✓	×	✓
CGTAEC	×	×	×
PDYSCKVFTGVYPFMWGGAYCFCD	×	×	✓
KFIVGPMSSAWTPFDNKIVVYKGDV	×	×	✓
YNMDYPPFGAGRPGQFGDIQSRTPE	✓	✓	×
SKDVYANTQLVLQRPAAGTVHVPYS	✓	✓	✓
QAPSGFKYWLKE	✓	✓	×
APFGCQIATNPVRAMNCAVGNMPIS	✓	×	✓
DIPDAAFTRVVDAP	✓		✓
FSTALAS	✓	×	✓

The algorithms used for prediction are Bepipred, Emini Surface Accessibility Area (ESA), Kolaskar and Tongaonkar (KT). Tick (✓) indicates that it is a predicted potential B cell epitope and cross (×) indicates that it is not a potential B cell epitope.

Out of the 24 conserved regions analyzed, 5 regions were predicted to be plausible B cell epitopes by all the three algorithms. 6 regions were predicted to be plausible B cell epitopes by at least 2 of the 3 algorithms tested. 12 regions were predicted to be B cell epitopes by only one of the algorithm. Only one out of the 24 conserved regions was not predicted as B cell epitope by all the three algorithms.

Interestingly, 9 out of the 24 conserved regions shared homology with human proteins which were experimentally proven to have antibodies developed against them in patients diagnosed with rheumatoid arthritis (highlighted in bold in Table 1).

**Figure 6:**
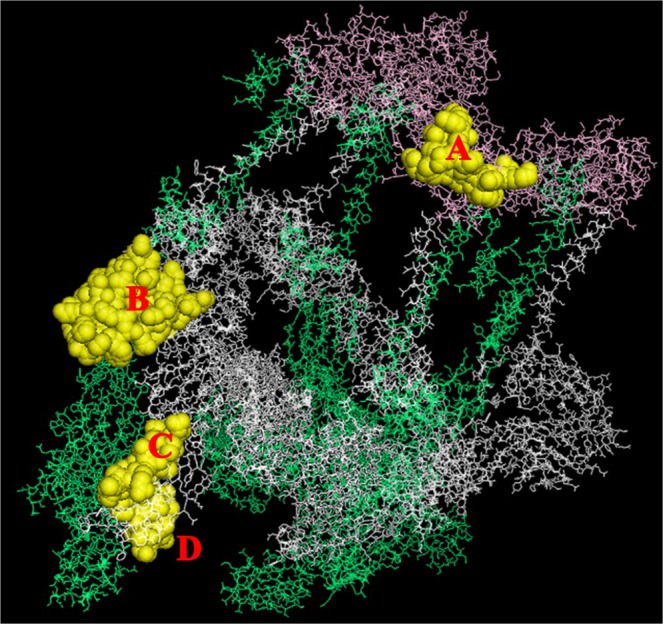
Epitopes predicted based on the 3D structure of the Chikungunya virus structural polyprotein (PDB ID: 3J2W) using ElliPro. The predicted peptides are shown in sphere representation and are colored yellow.

To further validate if the potential mimics could act as plausible B cell epitopes also at a conformational level, CHIKV structural polyprotein was used as a model to locate conformational B cell epitopes using Ellipro server from IEDB. As shown in Fig. 6, the parts of the peptides MKSDASKFTHEKPEGYYNWHHGAVQ and YSGGRFTIPTGAGKPGDSGRPIFDN (labelled A in Fig. 6) present on the Capsid protein, the part of the peptide RKGKIHIPFPLANVTCMVPKA (labelled B), PTVTYGK (labelled C) and EGLEVTWGNN (labelled D) present on the p62 protein are exposed to the surface and predicted to be potential conformational B cell epitopes.

## Discussion

Viral infection as one of the etiological agents for autoimmune diseases is being discussed for a long time. Several mechanisms have been proposed to explain this phenomenon including molecular mimicry, bystander activation and viral persistence^20^ individually or in multiple combinations to account for the immunopathology observed at the site of infection and/or sites of autoimmune disease and normally the infections precede the occurrence of inflammation in the target organ^21^. Triggered immune response as result of infection is critical for viral clearance. However, in some instances, immune regulatory mechanisms may aberrate, leading to the breakdown of self-tolerance, resulting in immune-mediated attack directed against both viral and self-antigens as it happens in the case of molecular mimicry or shared homology between viral and host epitopes^22,23^.

Human infections caused by the alphaviruses group share a common feature of the clinical picture of arthralgia and chronic arthritis that closely resembles rheumatoid arthritis^7,24,25^. Further, more severe and delayed recovery of alpha viral disease in patients with pre-existing arthritic conditions has been reported^26^. Though the cellular components and inflammatory scenario involved in such conditions have been looked at^27^, knowledge about the actual cause of such persistent symptoms characterized by articular disease and myalgia remains sparse. Though one of the speculations regarding the basis for this rheumatic like manifestations is the possible induction of autoimmunity as a side effect of adaptive immune responses, caused by cross-reactivity between viral and host antigens^24,28^, there are no systematic studies towards proving this hypothesis.

In the present work, through an *in silico* analysis we have identified the presence of conserved regions in the structural polyprotein of alphaviruses. A high sequence identity (71%) suggests that the origin of commonality in the clinical features may lie in the amino acid sequence of the structural polyprotein of the viruses. On comparative analysis of these regions of consensus with the human proteome, we have identified proteins that share stretches of homologous regions with the structural polyprotein. Further, very interestingly we found that presence of auto antibodies specific to some of these proteins in rheumatoid arthritis (Table 1). Serum and synovial fluid from RA patients have been shown to contain auto antibodies specific to some of the proteins that we identified in our analysis. These proteins are highlighted in table-1. The underlined region of the peptide SKDVYANTQLVLQRPAAGTVHVPYS has been experimentally tested in mice to induce polyarthralgia. The mouse injected with this peptide recapitulated the pathology seen chikungunya infection^29^. These reports give further credibility to our findings and strengthen our current premise.

**Figure 7:**
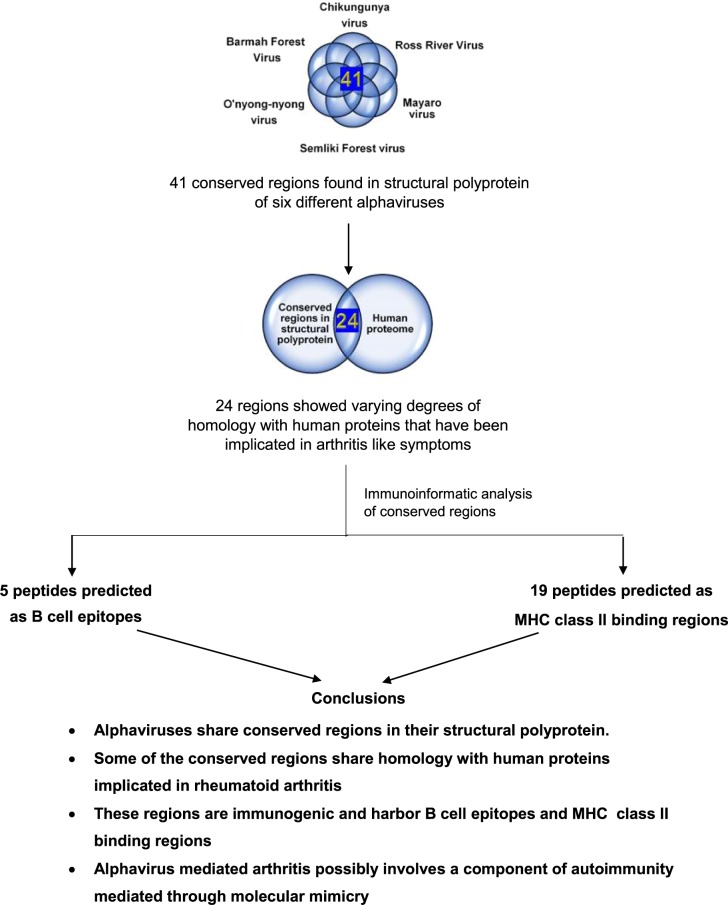
Highly conserved peptide sequences present in the structural polyprotein of alphaviruses are antigenic and could act as a trigger for autoimmune reactions explaining one of the possible origins for polyarthritis post-arthritogenic alphaviral infections.

Our results further show that these regions are immuno-dominant stressing their ability for induction of potential auto-immune reaction. Based on the amino acid sequence analysis, twenty-four of the conserved regions (Table 2) bound to HLA class II alleles, suggesting the possibility of calling in for T helper cell responses. Three conserved regions were predicted to be potential B cell epitopes by all the algorithms tested. Since majority of the antigenic epitopes have been shown to have high surface accessibility, we hypothesized that identical amino acids would give rise to similar structural features too. Therefore, we validated the antigenicity of chosen conserved regions at structural level. The root mean square deviation between the peptides is 0.86 Å which suggests that the two peptides bind to class II MHC in a similar orientation (Fig. 4). Docking the conserved region (FPLANVTCM) from the structural polyprotein and its homologous sequence in the human proteome revealed similar interactions with HLA-DRB1 (Fig. 5). Plausible B cell epitope prediction analysis based on the structure of CHIKV structural polyprotein concurred with some of the conserved regions we identified. Thus, validation at both the amino acid and structural level affirmed the antigenic potential of conserved regions in the structural polyprotein. In addition, through the revelation of presence of significant conserved regions in the alphaviral proteins, we surmise that antibodies for differential diagnosis of alpha virus infections should be directed towards the non-conserved regions of the structural proteins for higher specificity. In summary (Fig. 7), our results computationally tease out a possible mechanism mediated through molecular mimicry leading to development of autoimmunity during alpha virus infections which could culminate in arthritis like conditions in infected individuals.

## Methods

### Viral protein sequences

We carried out the bioinformatic analysis using protein sequences available for the clinical isolates of alphaviruses known to cause polyarthralgia^30–33^ and used as protype strains in the literature^29^. Structural polyproteins of the six arthritogenic alphaviruses: Chikungunya virus (strain S2-African prototype)(UniProt ID: Q8JUX5), the Ross river virus (strain NB5092) (UniProt ID: P13890), the Semliki forest virus (UniProt ID: P03315), the Mayaro virus (strain Brazil) (UniProt ID: Q8QZ72), the O’nyong-nyong virus (strain SG650) (UniProt ID: O90369) and the Barmah forest virus (UniProt ID: P89946) were investigated for the presence of conserved regions. Amino acid sequences of these proteins were retrieved from the UniProt database^34^.

### Multiple sequence alignment

Conserved regions within the structural polyproteins were identified by multiple sequence alignment using MUSCLE alignment algorithm^35^ in MEGA 7.0^36^. A stretch of amino acids was scored as conserved region if it is present in at least four out of the six alpha viruses.

### Homology search

BLASTp program (https://blast.ncbi.nlm.nih.gov/Blast.cgi)^37^ was employed to expound the existence of sequence homology between the conserved regions identified in the alpha viruses and the human proteome. The search set was limited to *Homo sapiens* (taxid: 9606) in the UniProtKB/Swiss-Prot database. Default BLASTp algorithm parameters were used and the results were limited only to the top 100 hits.

The homologous proteins list obtained from the BLAST search was manually curated using the Open Targets Platform server^38^ to identify if a given protein has been reported to be involved in the pathogenesis of rheumatoid arthritis. The Open Targets Platform server is a repository of human proteins and their involvement in diseases at various levels. These proteins bearing the homologous sequences to conserved regions within the structural polyproteins were chosen for further investigation.

### Prediction of potential T cell epitopes

The shortlisted conserved regions in the structural polyprotein were explored for their ability to act as T cell epitopes. Peptides from these regions were subjected to HLA II binding analysis using ProPred II analysis tool^39^. The server uses quantitative matrices for predicting binding of nonamers to HLA class II. Quantitative matrices are chosen for predictive binding because they provide a linear model and are easy to implement. Nonameric peptides generated from the conserved regions, which were predicted as potential binders for HLA class II alleles were selected for further analysis. A frequency distribution of the ability of these peptides to bind to multiple HLA class II alleles was generated.

Peptide-MHC docking was performed using GalaxyPepDock^40^ server to evaluate the similarity in binding patterns among a conserved region in the alphavirus structural polyprotein and its corresponding homologous region in the human protein. GalaxyPepDock works by identifying templates from experimentally resolved structure databases to predict the protein structure, followed by an energy-based optimization to provide structural flexibility. Default parameters available with the tool have been used and the back-end data containing the parameters used for docking are not available. Crystal structure of HLA-DRB1 complexed with Type II collagen peptide (PDB ID: 6BIN) was retrieved from Protein Data Bank database^41^. The resident peptide in the crystal structure was stripped off using PyMol to make the binding groove of HLA-DRB1 available for docking the viral peptide and its human homologue.

### Prediction of potential linear B cell epitopes

Shortlisted conserved regions from the structural polyprotein were explored for their ability to act as B cell epitopes. Full length amino acid sequence of structural polyprotein of Chikungunya virus (strain S27-African prototype) (UniProt ID: Q8JUX5) was subjected to linear B cell epitope prediction analysis available at Immune epitope database (IEDB)^42^. The analysis was performed using three different prediction algorithms namely, Bipipred, Emini surface accessibility and Kolaskar and Tongaonkar with default algorithm parameters.

Further, to validate if the conserved regions act as potential linear B cell epitopes at the structural level, we employed ElliPro server^43^ to predict the regions on the structural polyprotein of the Chikungunya virus (PDB ID: 3J2W). ElliPro accepts a PDB structure as input and uses three different algorithms based on protrusion index of the residues, protein shape approximation and neighboring residues clustering to predict linear antibody-based epitopes. Default parameters of the ElliPro program were used for prediction analysis.

## Supplementary information

Supplementary information

## Data Availability

All the viral sequences used for analysis were retrieved are from publicly available databases. Criteria used for analyzing and short listing peptide sequences have been explained clearly in materials and methods section. Most of the softwares used for HLA class II-peptide binding prediction and B cell epitope prediction are also available on the public domain and have been referenced accordingly. Protein structures used are from protein data bank and the relevant literature has been cited wherever appropriate. The other raw datasets generated during and/or analyzed during the study are available from the corresponding author on reasonable request. We declare that we will be fully willing to comply with the journal policy and will be able to make any materials/data available required for the review process and thereafter.
