# Supplementary material for: A possible role for autoimmunity through molecular mimicry in alphavirus mediated arthritis
Source: Sci Rep. 2020 Jan 22;10:938. doi: 10.1038/s41598-019-55730-6 (PMC6976597; doi:10.1038/s41598-019-55730-6)
Supplement: Supplementary file 1 — Supplementary information [file 41598_2019_55730_MOESM1_ESM.docx]

**A possible role for autoimmunity through molecular mimicry in alphavirus mediated arthritis**

**Siva Sai Krishna Venigalla, Sowmya Premakumar, Vani Janakiraman***

Department of Biotechnology, Bhupat and Jyoti Mehta School of Biosciences, Indian Institute of Technology Madras, Chennai 600036, India

**Fig. S8.** Multiple sequence alignment of structural polyproteins from six alphaviruses.

POLS_BFV 1 MDFIPTQTFYGRRWRPAP-VQRYIPQPQPPAP-------PRRRRGPSQLQ
POLS_CHIKS 1 MEFIPTQTFYNRRYQPRPWTPRPTIQVIRPRP--------RPQRQAGQLA
POLS_ONNVS 1 MEFIPAQTYYNRRYQPRPWTQRPTIQVIRPKP--------RRSRPAGQLA
POLS_MAYAB 1 MDFLPTQVFYGRRWRPRM-PPRPWRPRMPTMQ--------RPDQQARQMQ
POLS_RRVN 1 MNYIPTQTFYGRRWRPRP-AFRPWQVPMQPTPTMVTPMLQAPDLQAQQMQ
POLS_SFV 1 MNYIPTQTFYGRRWRPRP-AARPWPLQATPVA------PVVPDFQAQQMQ


POLS_BFV 43 QLVAALGALALQ--------PKQKQKRAQKKPKKTPPPKP--KKTQKPKK
POLS_CHIKS 43 QLISAVNKLTMR--AVPQQKPRRNRKNKKQKQKQQAPQNNTNQKKQPPKK
POLS_ONNVS 43 QLISAVSRLALR---TVPQKPRRTRKTKKQKQVKQEQQSTRNQKKKAPKQ
POLS_MAYAB 42 QLIAAVSTLALRQNAAA---PQRGKKKQPRRKKPKPQPEKPKKQEQKPKQ
POLS_RRVN 50 QLISAVSALTTKQNVKAPKGQRKQKQQKPKEKKEKQKKKPTXKKKQQQKP
POLS_SFV 44 QLISAVNALTMRQNAIAPARPPKPKKKKTTKPKPKTQPKKINGKTQQQKK


POLS_BFV 83 PTQK---KKSKPGKRMRNCMKIENDCIFPVMLDGKVNGYACLVGDKVMKP
POLS_CHIKS 91 KPAQ---KKKKPGRRERMCMKIENDCIFEVKHEGKVTGYACLVGDKVMKP
POLS_ONNVS 90 KQTQ---KKKRPGRRERMCMKIENDCIFEVKHEGKITGYACLVGDKVMKP
POLS_MAYAB 89 KKAP----KRKPGRRERMCMKIEHDCIFEVKHEGKVTGYACLVGDKVMKP
POLS_RRVN 100 KPQA---KKKKPGRRERMCMKIENDCIFEVKLDGKVTGYACLVGDKVMKP
POLS_SFV 94 KDKQADKKKKKPGKRERMCMKIENDCIFEVKHEGKVTGYACLVGDKVMKP


POLS_BFV 130 AHVKGTIDNPELAKLTFKKSSKYDLECAQVPVCMKSDASKFTHEKPEGHY
POLS_CHIKS 138 AHVKGTIDNADLAKLAFKRSSKYDLECAQIPVHMKSDASKFTHEKPEGYY
POLS_ONNVS 137 AHVKGTIDNADLAKLAFKRSSKYDLECAQIPVHMKSDASKFTHEKPEGYY
POLS_MAYAB 135 AHVPGVIDNADLARLSYKKSSKYDLECAQIPVAMKSDASKYTHEKPEGHY
POLS_RRVN 147 AHVKGTIDNPDLAKLTYKKSSKYDLECAQIPVHMKSDASKYTHEKPEGHY
POLS_SFV 144 AHVKGVIDNADLAKLAFKKSSKYDLECAQIPVHMRSDASKYTHEKPEGHY


POLS_BFV 180 NWHHGAVQFSNGRFTIPTGSGKPGDSGRPIFDNTGKVVAIVLGGANEGAR
POLS_CHIKS 188 NWHHGAVQYSGGRFTIPTGAGKPGDSGRPIFDNKGRVVAIVLGGANEGAR
POLS_ONNVS 187 NWHHGAVQYSGGRFTIPTGAGKPGDSGRPIFDNKGRVVAIVLGGANEGTR
POLS_MAYAB 185 NWHYGAVQYTGGRFTVPTGVGKPGDSGRPIFDNKGPVVAIVLGGANEGTR
POLS_RRVN 197 NWHHGAVQYSXGRFTIPTGAGKPGDSGRPIFDNKGRVVAIVLGGANEGAR
POLS_SFV 194 NWHHGAVQYSGGRFTIPTGAGKPGDSGRPIFDNKGRVVAIVLGGANEGSR


POLS_BFV 230 TALSVVTWNKDMVTRITPEESVEWSAAALNITALCVLQNLSFPCDAPPCA
POLS_CHIKS 238 TALSVVTWNKDIVTKITPEGAEEWSLA---IPVMCLLANTTFPCSQPPCI
POLS_ONNVS 237 TALSVVTWNKDIVTKITPEGSVEWSLA---LPVMCLLANTTFPCSQPPCA
POLS_MAYAB 235 TALSVVTWNKDMVTKITPEGTVEWAAST--VTAMCLLTNISFPCFQPSCA
POLS_RRVN 247 TALSVVTWTKDMVTRVTPEGTEEWSAAL--M--MCILANTSFPCSSPPCY
POLS_SFV 244 TALSVVTWNKDMVTRVTPEGSEEWSAPL--ITAMCVLANATFPCFQPPCV


POLS_BFV 280 PCCYEKDPAGTLRLLSDHYYHPKYYELLDSTMHCPQGRRPKRSVA-HFEA
POLS_CHIKS 285 PCCYEKEPEETLRMLEDNVMRPGYYQLLQASLTCSP-HRQRRSTKDNFNV
POLS_ONNVS 284 PCCYEKKPEETLRMLEDNVMQPGYYQLLDSALACSQ-HRQRRNARENFNV
POLS_MAYAB 283 PCCYEKGPEPTLRMLEENVNSEGYYDLLHAAVYCRNSSRSKRSTANHFNA
POLS_RRVN 293 PCCYEKQPEQTLRMLEDNVNRPGYYELLEASMTCRNRSRHRRSVIEHFNV
POLS_SFV 292 PCCYENNAEATLRMLEDNVDRPGYYDLLQAALTCRNGTRHRRSVSQHFNV


POLS_BFV 329 YKATRPYIGWCADCGLAGSCPSPVSIEHVWSDADDGVLKIQVSMQIGIAK
POLS_CHIKS 334 YKATRPYLAHCPDCGEGHSCHSPVALERIRNEATDGTLKIQVSLQIGIGT
POLS_ONNVS 333 YKVTRPYLAHCPDCGEGHSCHSPIALERIRSEATDGTLKIQVSLQIGIKT
POLS_MAYAB 333 YKLTRPYVAYCADCGMGHSCHSPAMIENIQADATDGTLKIQFASQIGLTK
POLS_RRVN 343 YKATRPYLAXCADCGDGYFCYSPVAIEKIRDEASDGMLKIQVSAQIGLDK
POLS_SFV 342 YKATRPYIAYCADCGAGHSCHSPVAIEAVRSEATDGMLKIQFSAQIGID

POLS_BFV 379 SNTINHAKIRYMGANGVQEAERSTLSVSTTAPCDILATMGHFILARCRPG
POLS_CHIKS 384 DDSHDWTKLRYMDNHIPADAGRAGLFVRTSAPCTITGTMGHFILARCPKG
POLS_ONNVS 383 DDSHDWTKLRYMDSHTPVDADRSGLFVRTSAPCTITGTMGHFILARCPKG
POLS_MAYAB 383 TDTHDHTKIRYAEGHDIAEAARSTLKVHSSSECTVTGTMGHFILAKCPPG
POLS_RRVN 393 AGTHAHTKMRYMAGHDVQESKRDSLRVYTSAACSIHGTMGHFIVAHCPPG
POLS_SFV 392 SDNHDYTKIRYADGHAIENAVRSSLKVATSGDCFVHGTMGHFILAKCPPG


POLS_BFV 429 SQVEVSLSTDPKLL--CRTPFSHKPRFIGNEKSPAPTGHKTRIPCKTYSH
POLS_CHIKS 434 ETLTVGFTDSRKISHSCTHPFHHDPPVIGREKFHSRPQHGKELPCSTYVQ
POLS_ONNVS 433 ETLTVGFVDSRRISHTCMHPFHHEPPLIGREKFHSRPQHGKELPCSTYVH
POLS_MAYAB 433 ERISVSFVDSKNEHRTCRIAYHHEQRLIGRERFTVRPHHGIELPCTTYQL
POLS_RRVN 443 DYLKXSFEDANSHVKACKVQYKHDPLPVGREKFVVRPHFGVELPCTSYQL
POLS_SFV 442 EFLQVSIQDTRNAVRACRIQYHHDPQPVGREKFTIRPHYGKEIPCTTYQQ


POLS_BFV 477 QTDLTREEITMHVPPDVPIQGLVSNTGKSYSLDPKTKTIKYKCTCGETVK
POLS_CHIKS 484 SNAATAEEIEVHMPPDTPDRTLLSQQSGNVKITVNGRTVRYKCNCGGS-N
POLS_ONNVS 483 TTAATTEEIEVHMPPDTPDYTLMTQQAGNVKITVDGQTVRYKCKCDGS-N
POLS_MAYAB 483 TTAETSEEIDMHMPPDIPDRTILSQQSGNVKITVNGRTVRYSSSCGSQ-A
POLS_RRVN 493 TTAPTDEEIDMHTPPDIPDRTLLSQTAGNVKITAGGRTIRYNCTCGRD-N
POLS_SFV 492 TTAETVEEIDMHMPPDTPDRTLLSQQSGNVKITVGGKKVKYNCTCGTG-N


POLS_BFV 527 EGTATNKITLFNCDTAPKCITYAVDNTVWQYNSQYVPR-SEVTEVKGKIH
POLS_CHIKS 533 EGLITTDKVINNC-KVDQCHAAVTNHKKWQYNSPLVPRNAELGDRKGKIH
POLS_ONNVS 532 EGLITTDKVINNC-KVDQCHTAVTNHKKWQYNSPLTPRNSEQGDRKGKIH
POLS_MAYAB 532 VGTTTTDKTINSC-TVDKCQAYVTSHTKWQFNSPFVPR-RMQAERKGKVH
POLS_RRVN 542 VGTTSTDKTINTC-KIDQCHAAVTSHDKWXFTSPFVPR-ADQTARKGKVH
POLS_SFV 541 VGTTNSDMTINTC-LIEQCHVSVTDHKKWQFNSPFVPR-ADEPARKGKVH


POLS_BFV 576 VPFPLTDSTCAVSVAPEPQVTYRLGEVEFHFHPMYPTLFSIRSLGKDPSH
POLS_CHIKS 582 IPFPLANVTCMVPKARNPTVTYGKNQVIMLLYPDHPTLLSYRSMGEEPNY
POLS_ONNVS 581 IPFPLVNTTCRVPKARNPTITYGKNRVTLLLYPDHPTLLSYRSMGRIPDY
POLS_MAYAB 580 IPFPLINTTCRVPLAPEALVRSGKREATLSLHPIHPTLLSYRTFGAERVF
POLS_RRVN 590 VPFPLTNVTCRVPLARAPDVTYGKKEVTLRLHPDHPTXFSYRSLGAVPHP
POLS_SFV 589 IPFPLDNITCRVPMAREPTVIHGKREVTLHLHPDHPTLFSYRTLGEDPQY


POLS_BFV 626 SQEWIDTPMSKTIQVGAEGVEYVWGNNNPVRLWAQKSSSSSAHGNPISIV
POLS_CHIKS 632 QEEWVTHKKEVVLTVPTEGLEVTWGNNEPYKYWPQLSANGTAHGHPHEII
POLS_ONNVS 631 HEEWITSKKEISITVPAEGLEVTWGNNDPYKYWPQLSTNGTAHGHPHEII
POLS_MAYAB 630 DEQWITAQTEVTIPVPVEGVEYQWGNHKPQRFVVALTTEGKAHGWPHEII
POLS_RRVN 640 YEEWVDKFSERIIPVTEEGIEYQWGNNPPVRLWAQLTTEGKPHGWPHEII
POLS_SFV 639 HEEWVTAAVERTIPVPVDGMEYHWGNNDPVRLWSQLTTEGKPHGWPHQIV


POLS_BFV 676 SHYYDLYPYWTITVLASLGLLIVISSGFSCFLCSVARTKCLTPYQLAPGA
POLS_CHIKS 682 LYYYELYPTMTVVVVSVASFILLSMVGMAVGMCMCARRRCITPYELTPGA
POLS_ONNVS 681 LYYYELYPTTTIAVLAAASIVVASLVSLSLGMCICARRRCITPYELTPGA
POLS_MAYAB 680 EYYYGLHPTTTIVVVIRVSVVVLLSFAASVYMCVVARTKCLTPYALTPGA
POLS_RRVN 690 QYYYGLYPAATIAAVSGASLMALLTLAATCCMLATARRKCLTPYALTPGA
POLS_SFV 689 QYYYGLYPAATVSAVVGMSLLALISIFASCYMLVAARSKCLTPYALTPGA

POLS_BFV 726 QLPTFIALLCCAKSARADTL-DDFSYLWTNNQAMFWLQLASPVAAFLCLS
POLS_CHIKS 732 TVPFLLSLICCIRTAKAATYQEAAVYLWNEQQPLFWLQALIPLAALIVLC
POLS_ONNVS 731 TIPFLLGVLCCVKTAKAASYYEAATYLWNEQQPLFWLQLLIPLSAAIVAC
POLS_MAYAB 730 VVPVTIGVLCCAPKAHAASFAEGMAYLWDNNQSMFWMELTGPLALLILAT
POLS_RRVN 740 VVPLTLGLLXCAPRANAASFAETMAYLWDENKTLFWMEXXXXXXALALLA
POLS_SFV 739 AVPWTLGILCCAPRAHAASVAETMAYLWDQNQALFWLEFAAPVACILIIT


POLS_BFV 775 YCCRNLACCMK--IFLGISGLCVIATQAYEHSTTMPNQVGIPFKALIERP
POLS_CHIKS 782 NCLRLLPCCCKTLAFLAVMSIGAHTVSAYEHVTVIPNTVGVPYKTLVNRP
POLS_ONNVS 781 NCLKLLPCCCKTLTFLAVMSIGARTVSAYEHATVIPNTVGVPYKTLVSRP
POLS_MAYAB 780 CCARSLLSCCKG-SFLVAMSIGSAVASAYEHTAIIPNQVGFPYKAHVARE
POLS_RRVN 790 CCIKSLICCCKPFSFLVLLSLGA-SAKAYEHTATIPNVVGFPYKAHIERN
POLS_SFV 789 YCLRNVLCCCKSLSFLVLLSLGA-TARAYEHSTVMPNVVGFPYKAHIERP


POLS_BFV 823 GYAGLPLSLVVIKSELVPSLVQDYITCNYKTVVPSPYIKCCGGAECSHKN
POLS_CHIKS 832 GYSPMVLEMELLSVTLEPTLSLDYITCEYKTVIPSPYVKCCGTAECKDKN
POLS_ONNVS 831 GYSPMVLEMELQSVTLEPTLFLDYITCEYKTITPSPYVKCCGTAECKAKN
POLS_MAYAB 829 GYSPLTLQMQVIETSLEPTLNLEYITCDYKTKVPSPYVKCCGTAECRTQD
POLS_RRVN 839 XFSPMTLQLEVVXXSLEPTLNLEYITCEYKTVVPSPFIKCCGTSECSSKE
POLS_SFV 838 GYSPLTLQMQVVETSLEPTLNLEYITCEYKTVVPSPYVKCCGASECSTKE


POLS_BFV 873 EADYKCSVFTGVYPFMWGGAYCFCDTENSQMSEVYVTRGESCEADHAIAY
POLS_CHIKS 882 LPDYSCKVFTGVYPFMWGGAYCFCDAENTQLSEAHVEKSESCKTEFASAY
POLS_ONNVS 881 LPDYNCKVFTGVYPFMWGGAYCFCDAENTQLSEAHVEKSESCKTEFASAY
POLS_MAYAB 879 KPEYKCAVFTGVYPFMWGGAYCFCDSENTQMSEAYVERADVCKHDHAAAY
POLS_RRVN 889 QPDYQCKVYTGVYPFMWGGAYCFCDSENTQLSEAYVDRSDVCKHDHALAY
POLS_SFV 888 KPDYQCKVYTGVYPFMWGGAYCFCDSENTQLSEAYVDRSDVCRHDHASAY


POLS_BFV 923 QVHTASLKAQVMISIGELNQTVDVFVNGDSPARIQQSKFILGPISSAWSP
POLS_CHIKS 932 RAHTASASAKLRVLYQGNNITVTAYANGDHAVTVKDAKFIVGPMSSAWTP
POLS_ONNVS 931 RAHTASVSAKLRVFYQGNNITVSAYANGDHAVTVKDAKFVIGPLSSAWSP
POLS_MAYAB 929 RAHTASLRAKIKVTYGTVNQTVEAYVNGDHAVTIAGTKFIFGPVSTPWTP
POLS_RRVN 939 KAHTASLKATIRISYGTINQTTEAFVNGEHAVNVGGSKFIFGPISTAWSP
POLS_SFV 938 KAHTASLKAKVRVMYGNVNQTVDVYVNGDHAVTIGGTQFIFGPLSSAWTP


POLS_BFV 973 FDHKVIVYRDEVYNEDYAPYGSGQAGRFGDIQSRTVNSTDVYANTNLKLK
POLS_CHIKS 982 FDNKIVVYKGDVYNMDYPPFGAGRPGQFGDIQSRTPESKDVYANTQLVLQ
POLS_ONNVS 981 FDNKIVVYKGEVYNMDYPPFGAGRPGQFGDIQSRTPDSKDVYANTQLILQ
POLS_MAYAB 979 FDTKILVYKGELYNQDFPRYGAGQPGRFGDIQSRTLDSRDLYANTGLKLA
POLS_RRVN 989 FDNKIVVYKDDVYNQDFPPYGSGQPGRFGDIQSRTVESKDLYANTALKLS
POLS_SFV 988 FDNKIVVYKDEVFNQDFPPYGSGQPGRFGDIQSRTVESNDLYANTALKLA


POLS_BFV 1023 RPASGNVHVPYTQTPSGFSYWKKEKGVPLNRNAPFGCIIKVNPVRAENCV
POLS_CHIKS 1032 RPAAGTVHVPYSQAPSGFKYWLKERGASLQHTAPFGCQIATNPVRAMNCA
POLS_ONNVS 1031 RPAAGAIHVPYSQAPSGFKYWLKEKGASLQHTAPFGCQIATNPVRAVNCA
POLS_MAYAB 1029 RPAAGNIHVPYTQTPSGFKTWQKDRDSPLNAKAPFGCIIQTNPVRAMNCA
POLS_RRVN 1039 RPSPGVVHVPYTQTPSGFKYWLKEKGSSLNTKAPFGCKIKTNPVRAMDCA
POLS_SFV 1038 RPSPGMVHVPYTQTPSGFKYWLKEKGTALNTKAPFGCQIKTNPVRAMNCA


POLS_BFV 1073 YGNIPISMDIADAHFTRIDESPSVSLKACEVQSCTYSSDFGGVASISYTS
POLS_CHIKS 1082 VGNMPISIDIPDAAFTRVVDAPSLTDMSCEVPACTHSSDFGGVAIIKYAV
POLS_ONNVS 1081 VGNIPVSIDIPDAAFTRVTDAPSVTDMSCEVASCTHSSDFGGAAVVKYTA
POLS_MAYAB 1079 VGNIPVSMDIADSAFTRLTDAPVISELTCTVSTCTHSSDFGGIAVLSYKV
POLS_RRVN 1089 VGSIPVSMDIPDSAFTRVVDAPAVTDLSCQVAVCTHSSDFGXVATLSYKT
POLS_SFV 1088 VGNIPVSMNLPDSAFTRIVEAPTIIDLTCTVATCTHSSDFGGVLTLTYKT

POLS_BFV 1123 NKVGKCAIHSHSNSATMKDSVQDVQESGALSLFFATSSVEPNFVVQVCNA
POLS_CHIKS 1132 SKKGKCAVHMTNAVTIREAEIEVEGNSQLQISFSTALASAEFRVQVCST
POLS_ONNVS 1131 SKKGKCAVHSLTNAVTIREPNVDVEGTAQLQIAFSTALASAEFKVQICST
POLS_MAYAB 1129 EKSGRCDIHSHSNVAVLQE--VSIETEGRSVIHFSTASASPSFVVSVCSS
POLS_RRVN 1139 DKPGKCAVHSHSNVATLQEATVDVKEDGKVTVHFSXXSASPAFKVSVCDA
POLS_SFV 1138 NKNGDCSVHSHSNVATLQEATAKVKTAGKVTLHFSTASASPSFVVSLCSA


POLS_BFV 1173 RITCHGKCEPPKDHIVPYAAKHNDAEFPSISTTAWQWLAHTTSGPLTILV
POLS_CHIKS 1182 QVHCAAECHPPKDHIVNYPASHTTLGVQDISATAMSWVQKITGGVGLVVA
POLS_ONNVS 1181 QVHCSATCHPPKDHIVNYPSPHTTLGVQDISTTAMSWVQKITGGVGLVVA
POLS_MAYAB 1177 RATCTAKCEPPKDHVVTYPANHNGVTLPDLSSTAMTWAQHLAGGVGLLIA
POLS_RRVN 1189 KTTCTAACEPPKDHIVPYGASHNNQVFPDMSGTAMTWVQRMASGLGGLAL
POLS_SFV 1188 RATCSASCEPPKDHIVPYAASHSNVVFPDMSGTALSWVQKISGGLGAFAI


POLS_BFV 1223 VAIIVVVVVSIVVCARH
POLS_CHIKS 1232 VAALILIVVLCVSFSRH
POLS_ONNVS 1231 IAALILIIVLCVSFSRH
POLS_MAYAB 1227 LAVLILVIVTCVTLRR-
POLS_RRVN 1239 IAVVVLVLVTCITMRR-
POLS_SFV 1238 GAILVLVVVTCIGLRR-
